# Immunomodulatory effect of *Tityus* sp. in mononuclear cells extracted from the blood of rheumatoid arthritis patients

**DOI:** 10.1590/1678-9199-JVATITD-2023-0064

**Published:** 2024-09-16

**Authors:** Cindy Gabriela Rivera Tobar, Yisel del Mar Morales Urmendiz, Marcela Alejandra Vallejo, Diego Felipe Manquillo, Victoria Eugenia Niño Castaño, Ana Isabel Ospina Caicedo, Leydy Lorena Mendoza Tobar, Jimmy Alexander Guerrero Vargas, Rosa Amalia Dueñas Cuellar

**Affiliations:** 1 Grupo de Investigación en Inmunología y Enfermedades Infecciosas, Programa de Medicina, Departamento de Patología, Facultad de Ciencias de la Salud, Universidad del Cauca, Popayán, Colombia.; 2 Grupo de Investigación en Salud, Programa de Medicina, Departamento de Medicina Interna, Facultad de Ciencias de la Salud, Universidad del Cauca, Popayán, Colombia.; 3 Grupo de Investigaciones Herpetológicas y Toxinológicas (GIHT), Departamento de Biología, Facultad de Ciencias Naturales, Exactas y de la Educación, Universidad del Cauca, Popayán, Colombia.; 4 Grupo de Investigaciones Herpetológicas y Toxinológicas (GIHT), Centro de Investigaciones Biomédicas - Bioterio (CIBUC-Bioterio), Museo de Historia Natural, Departamento de Biología, Facultad de Ciencias Naturales, Exactas y de la Educación, Universidad del Cauca, Popayán, Colombia.

**Keywords:** Rheumatoid arthritis, Venom, *Tityus* sp., Tityus aff. metuendus, Immunomodulatory activity, T lymphocytes, Proliferation, Activation, Flow cytometry

## Abstract

**Background:**

Pathophysiological mechanisms of rheumatoid arthritis arise because of a proinflammatory environment, generated by the interaction of autoreactive lymphocytes and proinflammatory mediators. Current strategies to mitigate the progression of the disease produce adverse effects, so there is a need for new therapeutic strategies and molecular targets to treat this disease. In this context, evidence suggests that scorpion venoms could modulate the immune response and some important cellular mechanisms of pharmacological interest. To evaluate the immunomodulatory effect of the venom of *Tityus* sp. (a possible new species close to *Tityus metuendus*) peripheral blood mononuclear cells of women diagnosed with RA were compared to cells of a control group.

**Methods:**

A case-control study was conducted with a sample of 10 women with a confirmed diagnosis of RA and controls matched by sex and age. The cytotoxicity of the venom was evaluated to find sublethal concentrations of the venom, and subsequently, their immunomodulatory capacity in terms of percentage of proliferation, cell activation, and cytokines production.

**Results:**

the venom of *Tityus* sp. produced a decrease in the percentage of proliferation in the CD3^+^, CD3^+^CD4^+^, and CD3^+^CD8^+^ cell subpopulations of RA patients and healthy controls, at concentrations of 252 and 126 µg/mL. However, the venom did not induce significant differences in the percentage of cell activation markers. The venom caused a decrease in IL-10 at a concentration of 252 µg/mL compared to untreated cells from patients and controls. The remaining cytokines did not show significant differences.

**Conclusion:**

the venom of *Tityus* sp. is a potential source of molecules with immunomodulatory ability in CD4 and CD8 T lymphocytes. This result directs venom characterization studies to identify pharmacological targets with immunomodulatory capacity in T lymphocytes to enhance research in the treatment of autoimmune disorders such as RA.

## Background

Rheumatoid arthritis (RA) is a global, chronic, inflammatory disease primarily affecting synovial membranes, leading to progressive damage to articular cartilage and bone. Autoreactive T and B cell activation triggers the release of pro-inflammatory cytokines (TNF-α, IL-1, IL-6, and IL-17), crucial in RA's pathophysiology [1-4]. The condition, affecting approximately 1% of adults worldwide, is more common in the 40-60 age group, with occasional cases in juveniles [5]. Currently incurable, treatment involves symptom control (e.g., glucocorticoids) and disease-modifying drugs (DMARDs) to manage progression, despite associated adverse effects [6-14].

In RA's pathophysiology, the persistent activation of autoreactive T lymphocytes in the synovial membrane sustains the activation of other cells like synovial macrophages and fibroblasts, transforming them into destructive cells. Studies suggest a crucial role of effector and memory T lymphocytes (TEM) CD4^+^CD45RA^−^CCR7^−^ in chronic inflammation, associated with proinflammatory cytokine production in the synovial membrane [15]. Simultaneously, potassium (K^+^) voltage-dependent channels (Kv) are vital in T lymphocyte activation, helping sustained cell activation through membrane hyperpolarization and calcium (Ca^+^) signaling, crucial for T cell differentiation [16, 17]. Significantly, Kv1.3 channel blockade has been linked to T lymphocyte inactivation and a subsequent reduction in effector cell function [16, 18].

Expression of Kv1.3 voltage-gated channels in T lymphocytes relies on differentiation state. In naive and central memory T cells, there are 400 to 500 Kv1.3 channels/cell, while effector and memory T cells (TEM) may express 1500 Kv1.3 channels/cell. This holds pharmacological interest given their role in autoimmune diseases and T cell activation [19]. The potassium channel KCa3.1, expressed at 10 channels/cell, also influences Ca+ signaling pathways in T cells. Differences in Kv1.3 channel numbers in TEM cells underscore their significance as potential therapeutic targets [17, 20]. As autoreactive TEMs contribute to autoimmune diseases, research focuses on finding Kv1.3 channel blockers as therapeutic targets for immunomodulating TEM cell responses in conditions like RA [17].

Potent potassium channel blockers, particularly peptides derived from scorpion venoms, have been extensively studied as potential immunomodulators. Casella Martins *et al*. [21], investigated the immunomodulatory effects of *Tityus serrulatus* venom at sub-lethal concentrations. Their findings revealed a decrease in T cell activation markers (CD69, CD25, and HLA-DR) and proliferation capacity, showing the immunomodulatory potential. Similar effects have been confirmed with various toxins from different scorpion species such as: a. Charibdotoxin, a peptide from the *Leiurus quinquestriatus hebraeus* venom [22, 23]; b. Iberiotoxin, a peptide from *Buthus tamulus* venom [24]; c. Kaliotoxin, a peptide from *Androctonus mauritanicus* venom [25]; d. Margatoxin peptide from *Centruroides margaritatus* venom [26]; e. OSK-1 (alpha-KTx3.7), a peptide from *Orthochirus scrobiculosus* venom [27]; and f. Vm24 (a-KTx 23.1), a peptide from *Vaejovis mexicanus smithi* venom [28]. All the above feature a Kv1.3 potassium channel blocking ability. 

Considering this, Hashemlou *et al.* [29], conducted an *in vivo* study in Wistar rats in which arthritis was induced and treated with *Mesobuthus eupeus* venom, showing that there was a significant reduction in the arthritis index score in all treated animals. In particular, a decrease in the size of the tibiotarsal joint region was evidenced in the groups that received crude scorpion venom and the control group (Betamethasone). Similarly, Tanner *et al.* [30], determined that Iberiotoxin (IbTX) from the *Buthus tamulus* scorpion can significantly reduce the severity of RA in Wistar rats, based on histological observations of immune infiltrates, pannus extensions, hyperplasia, and erosion of rat cartilage, without inducing significant side effects.

Additionally, Tanner *et al.* [19], in a Wistar rat model of RA, showed that the blockade of the KCa1.1 channel, using Iberiotoxin (IbTX) reduces the ability of synovial fibroblasts to stimulate the proliferation and migration of T_EM_ cells. Likewise, the blockade of the Kv1.3 channel by ShK-186/Dalazatide reduces the ability of T_EM_ cells to produce mediators that act on synovial fibroblasts, promoting the decrease in the expression of KCa1.1 molecules and major histocompatibility complex (MHC) class II in these cells. Furthermore, combination therapy of potassium channel blockers targeting KCa1.1 and Kv1.3 is more effective than monotherapies in reducing disease severity in RA rat models.

Considering the compelling evidence highlighting the impact of scorpion venom components on immune response modulation in both *in vitro* and *in vivo* models [31-33], Colombia, with approximately 81 scorpion species in its biodiversity, holds particular interest. The endemic species *Tityus* sp., a possible new species close to *Tityus metuendus* found in the state of Cauca, lacks comprehensive data on its venom's chemical composition and biological activity, making it a valuable subject for research. Morales *et al*. [34], used a venom gland cDNA library to identify toxins in *Tityus* sp. venom, including potassium channel-specific toxins from subfamilies α-KTx15, α-12, and α-KTx18. Furthermore, four toxins from this venom demonstrated the ability to reduce K+ current in rat dorsal ganglion cells *in vitro*.

Therefore, *Tityus* sp. venom is a promising source of toxins of pharmacological interest. Their immunomodulatory ability with potential use in the treatment of autoimmune diseases, however, has yet to be explored. Considering the above, in the present study the immunomodulatory effect of venom of *Tityus* sp. (VTsp) was evaluated on the percentage of proliferation (in the CD3^+^, CD3^+^CD4^+^, and CD3^+^CD8^+^ cell subpopulations) and activation percentage (in the CD4CD69^+^, CD4HLADR^+^, CD8CD69^+^, and CD8HLADR^+^) from peripheral blood mononuclear cells (PBMC) from female patients diagnosed with RA, compared with controls, as well as the presence of cellular mediators (IL-1β, IL-6, IL-10, and TNF-α ) in culture supernatants.

## Methods

### Venom collection and preparation

50 adults of *Tityus* sp. scorpions (male and female) were collected in the city of Popayán. The specimens were taken to the *Centro de Investigaciones Biomédicas* - vivarium (CIBUC) of the Universidad del Cauca where they were provided with food and water *ad libitum*. The VTsp was extracted using electrostimulation (Lafayette instruments) [35] at 30 V direct current, lyophilized (FreeZone 2.5 - LABCONCO, USA), and stored at -70 °C until its use.

The lyophilized venom was diluted with 200 µL of 1X phosphate-buffered saline (PBS) (Gibco/Invitrogen, Van Allen Way, Carlsbad, CA, USA) then homogenized (Heidolph, USA) and subsequently transferred to a sterile 15 mL tube (Falcon BD, Franklin Lakes, NJ, USA). Quantification of the complete venom was performed by reading at 280/260 nm in a microplate spectrophotometer (Multiskan SkyHigh Photometer - Thermo Fisher Scientific, USA).

### Isolation and culture of peripheral blood mononuclear cells (PBMC)

PBMCs were isolated from blood taken by venipuncture, by the discontinuous density centrifugation method via Ficoll-Hypaque (Hystopaque®-1077; Sigma Aldrich, USA). The PBMCs were washed twice and resuspended in RPMI 1640 medium (Gibco, USA). 150,000 cells/200 μL were cultured per well in a 96-well microplate (Fisher Scientific, USA) in RPMI 1640 medium (Gibco, USA) supplemented with 10% fetal bovine serum (Gibco, USA), 1% Penicillin/Streptomycin (BioWhittaker, USA), and stimulated with Phytohemagglutinin (PHA; 2.5 µg/mL; Sigma-Aldrich, USA) in the presence or otherwise of different concentrations of *Tityus* sp. venom at 37 °C and 5% CO_2_, for 72 hours. 

### **Evaluation of the cytotoxic activity of *Tityus* sp. venom on PBMC and determination of inhibitory concentration 50 (IC**_50_ **)**

The VTsp cytotoxicity test was performed using the resazurin method [36]. PBMC from healthy donors, under culture conditions described above, were exposed to different concentrations of the *Tityus* sp. venom (2000; 1000; 500; 250; 125; 62.5; 31.2; 15.6; 7.8 and 3.9 µg/mL) for 72 hours at 37 °C and 5% CO_2_. Untreated cells were used as the negative control and Triton 1X (Sigma-Aldrich, USA) as a positive control. 16 hours before the end of the culture time, resazurin (Alamar-Blue; Invitrogen, USA) was added at 10% v/v in each well. After 72 hours of culture, readings were done at 570 nm and 630 nm in a microplate spectrophotometer (Multiskan SkyHigh Photometer - Thermo Fisher Scientific, USA). The percentage reduction in cell viability was calculated based on the oxidation/reduction formula [37]. The effect of the venom was expressed in percentages of cell viability concerning the negative control.

Determination of the IC_50_ was established as the concentration in which cell viability was reduced by 50%. It was calculated using GraphPad Prism 8 software, taking as variables the concentration of venom, mortality, and control groups. From the IC_50_ value, three sublethal concentrations were taken that corresponded to 30, 15, and 7.5% of the IC_50_ value, respectively; considering a percentage of viability greater than 80% for PBMC, such that cell integrity could be ensured in the 72 hours of culture.

### Recruitment of patients with RA and social and clinical profile analysis

Ten females diagnosed with RA (18 to 69 years old) from the State of Cauca, were recruited from the private consultation of rheumatologist Ana Isabel Ospina, as defined in the criteria for the diagnosis of rheumatoid arthritis provided by the European League Against Rheumatism (EULAR) and the American College of Rheumatology (ACR) in 2010 [38, 39]. Additionally, ten healthy females were recruited as control and matched by age and sex with the patients. The exclusion criteria included not presenting symptoms referring to any acute or chronic disease, and not having consumed any non-steroidal anti-inflammatory drugs (NSAIDs) and/or disease-modifying drugs (DMARDs) for at least four months. Both patients and controls were interviewed using a sociodemographic survey. All study participants signed informed consent, in accordance with the ethics endorsement issued by the Universidad del Cauca (ID-4783).

### Immunophenotyping of lymphocyte subpopulations and activation markers

Immunophenotyping was performed using flow cytometric analysis to identify TCD4^+^ and TCD8^+^ lymphocyte subpopulations and measure levels of activation markers in PBMC from RA patients as controls. For this, the cells (5x10^6^ cells per well) that were seeded in the presence or otherwise of VTsp (252; 126; 63 µg/mL) for 72 hours at 37 °C and with 5% CO_2_, were collected and washed with 200 µL of buffer (1X PBS; Gibco/Invitrogen, Van Allen Way, Carlsbad, CA, USA) and labeled with anti-CD4-APC and anti-CD8-PECy7; and anti-CD69-FITC and anti-HLA-DR-PE antibodies; (BD Bioscience, USA). The cells were then incubated for 30 minutes in the dark. Two washes were performed and resuspended in 1x PBS.

For the flow cytometry analysis, a total of 20,000 events were acquired in the flow cytometer (Accuri C6 flow cytometer - BD Biosciences, USA). The analysis strategy was carried out as follows: the gate for the lymphocytes was selected according to the forward and side scatter distribution and the percentage of CD4^+^CD69^+^, CD4^+^ HLA-DR^+^, CD8^+^CD69^+^, and CD8^+^ HLA-DR^+^ cells were identified using a Dotplot graph.

### Evaluation of cell proliferation

For the cell proliferation test, the staining protocol with 5(6)-Succinimidyl Ester Diacetate of Carboxyfluorescein/CFSE (Molecular Probes/Invitrogen, Van Allen Way, Carlsbad, CA, USA), modified from Lyons [40], was performed. About 5x10^6^ cells/mL were collected in 15 mL tubes. They were then washed and resuspended in 1X PBS to perform the labeling with CFSE (0.062 µM; Molecular Probes, USA) for 15 minutes at 37 °C. Two washes were then performed; the first with 1X PBS and the second with complete RPMI 1640 medium. The cells were then resuspended in RPMI 1640 complete medium and incubated for 30 minutes at 37 °C.

The PBMCs previously labeled with CFSE were cultured in a 96-well microplate (Fisher Scientific, USA) in RPMI 1640 complete medium (Gibco, USA) supplemented with 10% fetal bovine serum (Gibco, USA), 1% Penicillin/Streptomycin (BioWhittaker, USA), and stimulated with Phytohemagglutinin (PHA; 2.5 µg/mL; Sigma-Aldrich, USA) in the absence or otherwise of *Tityus* sp. venom (252, 126 and 63 µg/mL) at 37 °C, under 5% CO_2_, for 72 hours. Then, the cells were labeled with anti-CD3-PE, anti-CD4-APC, and anti-CD8-PECy7 monoclonal antibodies (BioLegend, USA) and incubated for 30 minutes in the dark. Reading by flow cytometry (Accuri C6 flow cytometer - BD Biosciences, USA).

The analysis strategy to identify the percentage of cells in proliferation was carried out as follows: the region of the lymphocytes was identified through a gate, considering forward and side scatter, and using a Dotplot graph and histogram, the percentage was quantified of CFSE^low^ lymphocytes in the CD3^+^, CD3^+^CD4^+^, and CD3^+^CD8^+^ subpopulations.

### Cytokine quantification

The cytokines IL-6, TNF-α, IL1-β, and IL-10 were quantified by enzyme-linked immunosorbent assay (ELISA) using ELISA MaxTM Deluxe Set kits (BioLegend®, San Diego, CA 92121, USA) from PBMC culture supernatants (72 hours; 37 °C; 5% CO_2_), treated with PHA (2.5 µg/mL) in the presence or otherwise of VTsp (252; 126; 63 µg/mL). The procedure was conducted according to manufacturer specifications and the reading was carried out in the EPOCH 2 spectrophotometer (Agilent Biotek, USA).

### Statistical analysis

The results were analyzed using a two-way analysis of variance (ANOVA), considering a significance value of p < 0.05, followed by the Tukey multiple comparison test. Bar graphs were prepared in which the mean and the standard error of the mean (± SEM) were expressed for each condition and the sociodemographic analysis of the patients with RA and healthy controls were analyzed using bivariate analysis in GraphPad Prism 8 statistical analysis software.

## Results

### Determination of inhibitory concentration 50 and sublethal concentrations of *Tityus* sp. venom

**Figure 1.**
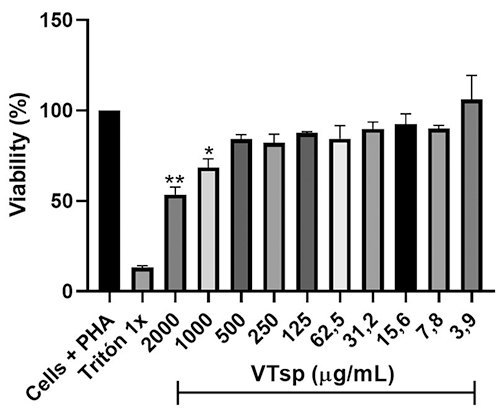
Effect of *Tityus* sp. venom (VTsp) on the cell viability of peripheral blood mononuclear cells (PBMC), that were stimulated with PHA (Phytohemagglutinin), and exposed to the VTsp in a 72h culture, using the resazurin fluorimetric assay (AlamarBlue). Negative control: Triton 1x; positive control: untreated cells. Results were expressed as the standard error of the mean (± SEM). Significance values p < 0.05* and p < 0.01**.

The percent cell viability in the presence of the different concentrations of venom was reduced on a dose basis depending on the concentrations of VTsp used compared to the control (untreated cells), with 2000 and 1000 µg/mL being the concentrations with the greatest percent drop in viability. The IC_50_ calculation yielded a concentration equivalent to 840.3 µg/mL (Figure 1). Considering the above, concentrations 252, 126, and 63 µg/mL were selected; corresponding to 30, 15, and 7.5% of the IC_50_ value, as sublethal concentrations to conduct the study of immunological variables.

### Social and clinical profile analysis of AR patients and controls

In the sociodemographic analysis, it was found that the ten patients with RA (n = 10: 100%) had an average age of 41 ± 0.51 years, and the age range was 20-69 years. The most recurrent occupations among RA patients were student (n = 2: 20%), housewife (n = 3: 30%), independent (n = 2: 20%), and professionals (n = 3 30%). Additionally, the clinical findings show that RA patients suffer pain and/or swelling in large joints where 90% (n = 9) reported in the knee, followed by shoulders (n = 8: 80%), ankles (n = 8: 80%), hips, and elbows (n = 7: 70%) and in small joints, where 100% (n = 10) of the patients had problems in the metacarpophalangeal joints, as well as wrists (n = 9: 90%), proximal interphalangeal joints (n = 8: 80%) and metatarsophalangeal joints (n = 3: 30%).

### The *Tityus* sp. venom did not induce changes in the percentage of PBMC activation markers

**Figure 2.**
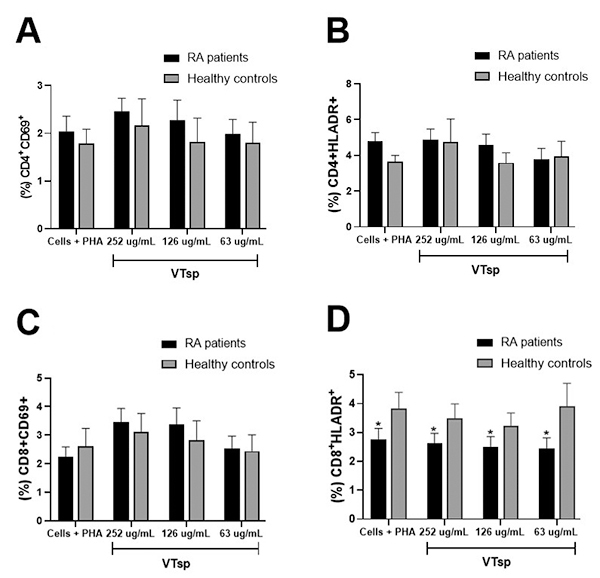
Percentage of cell activation of peripheral blood mononuclear cells (PBMC) that were stimulated with PHA (Phytohemagglutinin), and exposed to the VTsp, in a 72h culture. Activation analysis of **(A)** CD4^+^CD69^+^, **(B)** CD4^+^HLADR^+^, **(C)** CD8CD69^+^, **(D)** CD8^+^HLADR^+^, cell populations exposed to *Tityus* sp. venom (252, 126, and 63 µg/mL) comparing RA patients and healthy controls.

The subpopulations of CD4^+^ and CD8^+^ T cells from patients with RA and healthy controls, stimulated with PHA and treated or otherwise with *Tityus* sp. venom, did not show significant changes in the percentage of expression of the activation markers CD69^+^ and HLADR^+^. Comparing the activation markers, regardless of treatment, it was seen that the percentage of CD8^+^ HLADR^+^ cells was lower in RA patients compared to controls, with a statistical difference of p < 0.05 (Figure 2).

### ***Tityus* sp. venom induced a decrease in the percentage of proliferation of the subpopulations of CD4** ^+^ **and CD8** ^+^ **T lymphocytes**

**Figure 3.**
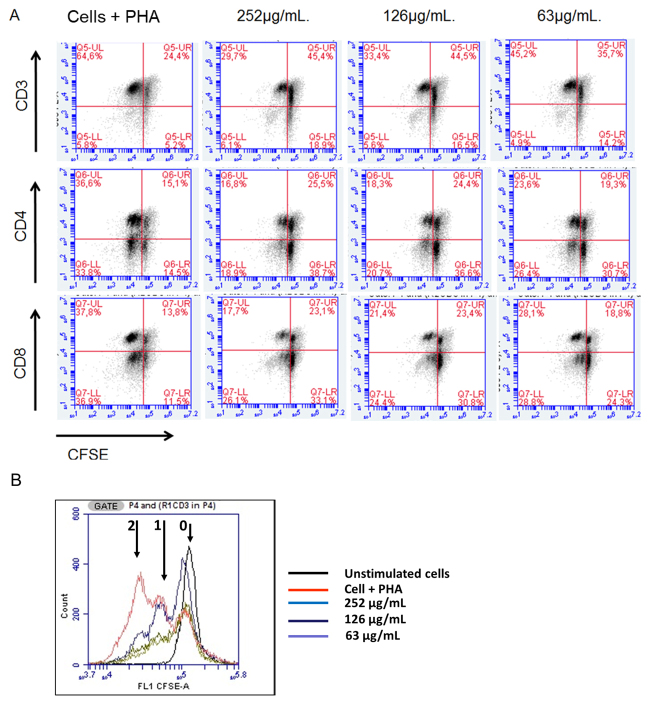
Effect of *Tityus* sp. venom on the percentage of PBMC proliferation; **(A)** Dotplot representative of the cytometric analysis to assess the percentage of cell proliferation in the CD3^+^, CD3^+^CD4^+^, CD3^+^CD8^+^ populations that were stimulated with PHA (Phytohemagglutinin), marked with CFSE, and exposed to *Tityus* sp. venom in a 72-hour culture; **(B)** Representative histogram of the effect of *Tityus* venom on PBMC proliferation. The arrows refer to the number of cell cycles that occurred during the 72 hours.

**Figure 4.**
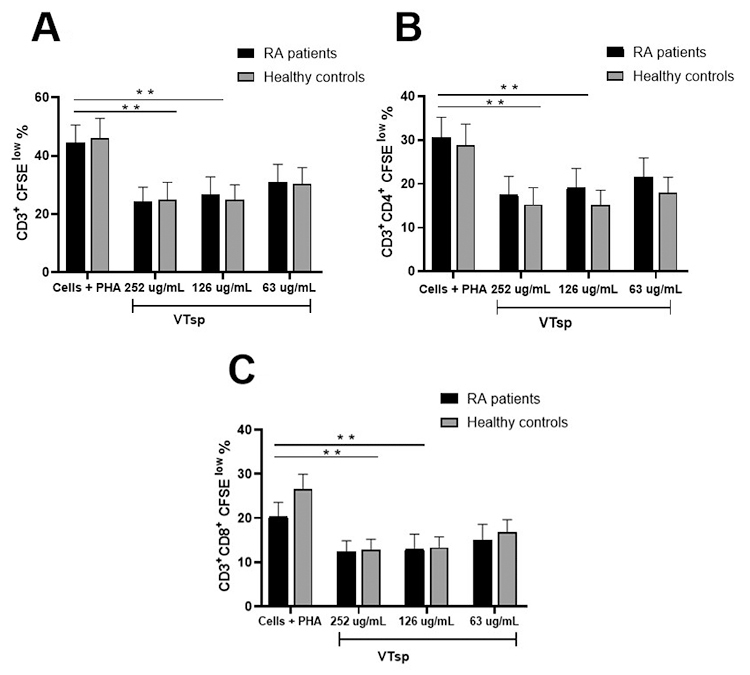
Percentage of the proliferation of PBMC treated with *Tityus* sp. venom. Proliferation analysis of **(A)** CD3^+^, **(B)** CD3^+^CD4^+^, **(C)** CD3CD8^+^ CFSE^low^ cell populations, which were stimulated with PHA (Phytohemagglutinin) and exposed to *Tityus* sp. venom (252, 126 and 63 µg/mL) comparing RA patients and healthy controls. Results are expressed as mean standard error (± MSE). Significance value p < 0.01**.

On evaluating the immunomodulatory capacity of the three concentrations of *Tityus* sp. venom regarding cell proliferation, the statistical analysis showed a significant difference (p < 0.05). The multiple comparison analysis showed a significant decrease between proliferation percentages for CFSE^low^ cell subpopulations in CD3^+^, CD3^+^CD4^+^, and CD3^+^CD8^+^ lymphocytes, in the untreated condition compared to cells treated with the concentrations 252 µM/mL and 126 µM/mL of the venom (p < 0.01) (Figures 3 and 4).

### *Tityus* sp. venom induced a decrease in the release of cytokine IL-10

**Figure 5.**
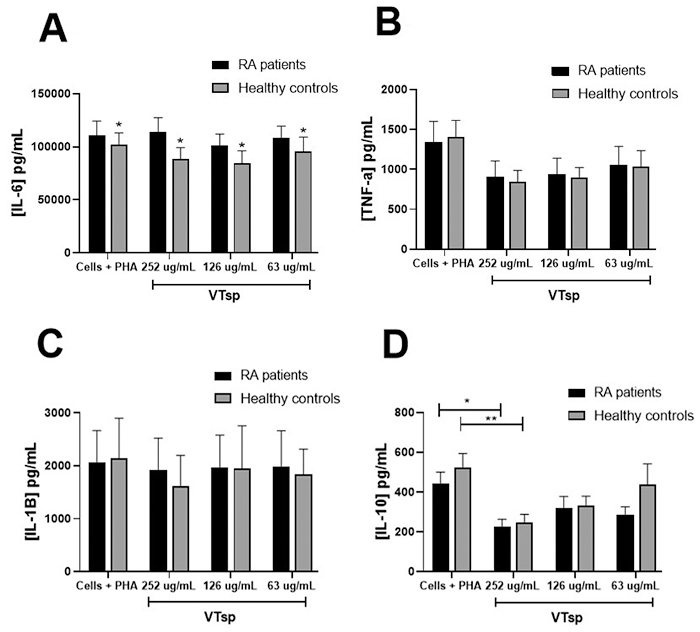
Analysis of cytokine expression of PBMC from RA patients and healthy controls exposed to VTsp venom. Production of cytokines **(A)** IL-6, **(B)** TNF-α, **(C)** IL1-β, and **(D)** IL-10 expressed in pg/mL of mononuclear cells from patients diagnosed with RA and healthy patients cultured with PHA and treated with *Tityus* sp. Results are expressed as the mean standard error (± MSE). Significance values were p < 0.01** and p < 0.05*.

On analyzing the expression of the cytokines IL-6, TNF-α, IL1-β, and IL-10 (Figure 5), a significant decrease in the level of cytokine IL-10 was observed in the cells treated with 252 µg/mL of the venom compared to untreated cells in RA patients and healthy controls (Figure 5 D ). No significant differences were observed in the concentration of IL-6, TNF-α, and IL1-β, in any of the conditions evaluated. In the comparison of the levels of cytokines between patients and controls and independently of the experimental conditions, a significant increase in the level of the cytokine IL-6 was found in RA patients compared to healthy controls (p < 0.05) (Figure 5 A ).

## DISCUSSION

The present study evaluated the immunomodulatory activity of the venom of the *Tityus* sp. scorpion on the ability for cell proliferation and activation in populations of CD8^+^ and CD4^+^ T lymphocytes, from patients diagnosed with RA and controls, as well as the production of cytokines IL-6, TNF-α, IL1-β, IL-10, in 72h cell culture supernatants. The results obtained show that the venom-induced significant changes and responses regarding cell proliferation and cytokine secretion independently of the state of disease.

Determination of the IC_50_ of *Tityus* sp. venom *in vitro* assays was crucial to assess its toxicity and conduct more controlled research processes, mainly considering that, in the case of this research, it was necessary to start from cells with viability greater than 80% to measure the functional conditions of immune response cells. To our knowledge, to date, this is the first report of IC_50_ for *Tityus* sp. venom *in vitro* assays.

The results of the present study did not show differences in the percentage of cells that express CD69 and HLA-DR between the different treatments carried out on PBMC of RA patients when compared with the treatments carried out on the PBMC of healthy controls. However, when the percentage of CD8^+^ HLADR^+^ cells was compared between the groups analyzed, it was found that in RA patients the percentage was lower compared to controls, indicating that the lower percentage of CD8^+^HLADR^+^ cells is associated with the disease and not with the treatment with the different concentrations of the venom. These can be explained by studies that show correlations between the severity of RA and the number and phenotype of CD8^+^ T cells in peripheral blood, or in inflammatory tissue [41]. Other research mentions that, in patients with early RA, the absolute number of CD8^+^ T cells is higher than in healthy controls; while in patients in remission, the number of CD8^+^ T lymphocytes is reduced [42].

We can recall that VTsp venom is composed of, among other molecules, specific toxins for potassium channels classified in the α-KTx15, α-12, and α-KTx18 subfamilies, capable of decreasing the K+ current in rat dorsal ganglion cells *in-vitro* [34], As such, about the proliferation results, the significant decrease between proliferation percentages for CFSE^low^ cell subpopulations in CD3^+^, CD3^+^CD4^+^ and CD3^+^CD8^+^ lymphocytes in untreated condition compared with cells treated with venom concentrations of 252 µg/mL and 126 µg/mL may be related to the fact that certain components of the venom may be highly selective for Kv1.3 channels. This relates to the modulation of the cellular response of the T lymphocytes [43] since it has been shown that the selective blockade of K+ channels (mainly Kv1.3) leads to depolarization of the membrane and inhibits the influx of Ca^2+^, resulting in a diminution of cell proliferation and of its effector functions [19, 44].

Considering this, the study by Varga *et al.* [45] demonstrated that the Vm24 toxin, from the scorpion *Vaejovis mexicanus smithi*, inhibited the proliferation of T lymphocytes stimulated with anti-CD3/CD28, after 96h of culture in all the concentrations evaluated (100, 50, 10, 5, 1 and 0.1 pM; likewise, the toxin inhibited the expression of the CD25 marker in stimulated T CD4^+^ lymphocytes. In addition, the authors verified that Vm24 can inhibit the Kv1.3 channels of human lymphocytes with high affinity, with a selectivity 1500 times greater than other channels such as KCa3.1, Kv1.1, and Kv1.2; as well as reducing delayed-type hypersensitivity (DTH) reactions in *in-vivo* models.

Additionally, it is noteworthy that since Kv.1.3 is a predominant potassium channel in resting T cells (naïve or T_EM_), proliferation is sensitive to inhibition of this channel, however, it is not complete, which may be an indicator of the presence of other channels in cells [45-47].

The obtained results also correlate with those obtained by Casella Martins *et al*. [21], who reported a decrease in the percentage of proliferation in populations of PBMC CD4^+^CD25^+^ T lymphocytes treated with *Tityus serrulatus* venom (concentrations of 50 and 100 µg/mL), compared to the control (cells + PHA). Ion channels are known to be involved in the early phase of lymphocyte activation by Phytohemagglutinin (PHA). Since PHA is a mitogen that acts by activating T lymphocytes by binding to the TCR receptor, it is possible that the toxins present in *Tityus serrulatus* venom blocked the K+ channels of CD4^+^CD25^+^ cells and impaired PHA action and consequently the activation percentage, as in this study [16, 21].

The *Tityus* sp. venom, as demonstrated in the chromatographic profiles obtained by Duque-Morales [34], and Arenas [48], has a profile that follows the pattern reported by Barona *et al*. [49], for *Tityus pachyurus* and Guerrero Vargas *et al*. [50] for *Tityus obscurus*, showing the region from 10 to 30 min (retention time) with fractions corresponding to toxins that act on potassium channels, therefore, it is possible to infer that the venom of *Tityus* sp. may have one or more toxins that act on Kv1.3 potassium channels. Based on this inference, and with the results obtained in this study, the venom of *Tityus* sp present a pharmacological and biotechnological potential as demonstrated in other closed species of scorpions [51]. 

Specifically, Kv1.3 potassium channels are considered a new therapeutic target to treat and diagnose inflammatory diseases, given their crucial role in T lymphocytes [52]. Based on the results obtained in this work, the venom of *Tityus* sp. and its toxins with action on Kv1.3 could be promising immunomodulatory molecules that can be used in treatments for autoimmune diseases, an aspect consistent with Zhao *et al*. [53] and Harunur *et al.* [54].

Investigations have shown that some scorpion toxins and complete venoms can inhibit certain proinflammatory cytokines and/or enhance the production of anti-inflammatory cytokines such as the IL-10 cytokine, both in *vivo* and *ex vivo* [32, 55, 56]. However, contrary to these findings, in the present study a decrease in the concentration of IL-10 was obtained in PBMC culture supernatants exposed to a concentration of 252 µg/mL of *Tityus* sp. venom, both in patients with RA and healthy patients. This result matches with the study of Veytia-Buchelli *et al.* [57], who treated T_EM_ CD4^+^ lymphocytes isolated from healthy donors with the Vm24 toxin from the *Vaejovis mexicanus* scorpion and found a decrease in the production of this cytokine, consistent with an inhibition of the transcription factor NFATc2 (Nuclear factor of activated T cells) and the recruitment of IRF4 (Interferon regulatory factor 4); two transcription factors involved in T cell activation, proliferation, and differentiation, which synergistically increase the activity of the Th2-specific enhancer, CNS-9 (a regulatory element downstream of the IL-10 gene locus).

Finally, the increase in IL-6 in patients with RA compared to healthy controls coincides with the results obtained by Wang *et al.* [58], who report an increase in IL-6 levels obtained from PBMC culture supernatants of 24h, stimulated with PHA in patients with RA compared to healthy controls. Usually, this cytokine is associated with inflammatory processes and is found to be in abundance in the synovial fluid and the serum of patients with RA. The increase in its levels has been correlated with the activity and progression of the disease and joint destruction [59], therefore, their increased presence in culture supernatants from people with RA is synonymous with the inflammatory processes characteristic of the disease.

## CONCLUSIONS

The results of this study showed a strong suggestion of an immunomodulatory effect induced by *Tityus* sp. venom on PBMCs, both in patients with RA and in healthy patients, mainly in cell proliferation (concentrations of 252 and 126 µg/mL) and in the decrease of cytokines such as IL-10, possibly associated with the presence of toxins that act on ionic channels, mainly potassium K+. IL-6 cytokine levels were higher in RA patients compared to healthy controls, confirming the inflammatory state associated with the disease.

Considering that this study shows the immunomodulatory potential of the venom of the *Tityus* sp. scorpion, the need to carry out detailed investigations of its components is highlighted to identify which ones participate in the process of modulating the response of T lymphocytes, that, in the context of rheumatoid arthritis, as well as in other autoimmune diseases, has a major role in pathophysiology. As such, the study of new therapeutic alternatives for RA is a vital field in which the prospective research of the venom of this species acquires relevance as a possible therapeutic agent.

## Data Availability

The datasets generated during and/or analyzed during the current study are available from the corresponding author upon reasonable request.

## References

[B1] McInnes IB, Schett G (2011). Mechanism of Disease The Pathogenesis of Rheumatoid
Arthritis. N Engl J Med.

[B2] Scott IC, Machin A, Mallen CD, Hider SL (2018). The extra-articular impacts of rheumatoid arthritis: moving
towards holistic care. BMC Rheumatol.

[B3] Alunno A, Carubbi F, Giacomelli R, Gerli R (2017). Cytokines in the pathogenesis of rheumatoid arthritis: new
players and therapeutic targets. BMC Rheumatol.

[B4] Croia C, Bursi R, Sutera D, Petrelli F, Alunno A, Puxeddu I (2019). One year in review 2019: pathogenesis of rheumatoid
arthritis. Clin Exp Rheumatol.

[B5] Kvien TK, Uhlig T, Ødegård S, Heiberg MS (2006). Epidemiological Aspects of Rheumatoid Arthritis: The Sex
Ratio. Ann N Y Acad Sci.

[B6] Smolen JS, Landewé R, Bijlsma J, Burmester G, Chatzidionysiou K, Dougados M, Nam J, Ramiro S, Voshaar M, van Vollenhoven R, Aletaha D, Aringer M, Boers M, Buckley C, Buttgereit F, Bykerk V, Cardiel M, Combe B, Cutolo M, van Eijk-Hustings Y, Emery P, Finckh A, Gabay C, Gomez-Reino J, Gossec L, Gottenberg JE, Hazes JM, Huizinga T, Jani M, Karateev D, Kouloumas M, Kvien T, Li Z, Mariette X, McInnes I, Mysler E, Nash P, Pavelka K, Poór G, Richez C, van Riel P, Rubbert-Roth A, Saag K, da Silva J, Stamm T, Takeuchi T, Westhovens R, de Wit M, van der Heijde D (2013). EULAR recommendations for the management of rheumatoid arthritis
with synthetic and biological disease-modifying antirheumatic drugs: 2013
update Josef. Ann Rheum Dis.

[B7] Aletaha D, Alasti F, Smolen JS (2016). Optimisation of a treat-To-Target approach in rheumatoid
arthritis: Strategies for the 3-month time point. Ann Rheum Dis.

[B8] Lopez-Olivo MA, Tayar JH, Martinez-Lopez JA, Pollono EN, Cueto JP, Gonzales-Crespo MR, Fulton S, Suarez-Almazor ME (2012). Risk of malignancies in patients with rheumatoid arthritis
treated with biologic therapy: A meta-analysis. JAMA.

[B9] Kremer JM, Romain PL (2020). Major side effects of low-dose methotrexate. UpToDate.

[B10] Fox R, Helfgott SM (2019). Pharmacology, dosing, and adverse effects of leflunomide in the
treatment of rheumatoid arthritis. UpToDate.

[B11] Linares V, Alonso V, Domingo JL (2011). Oxidative stress as a mechanism underlying sulfasalazine-induced
toxicity. Expert Opin Drug Saf.

[B12] Fleischmann R, van Vollenhoven RF, Vencovský J, Alten R, Davies O, Mountian I, de Longueville M, Carter D, Choy E (2017). Long-Term Maintenance of Certolizumab Pegol Safety and Efficacy,
in Combination with Methotrexate and as Monotherapy, in Rheumatoid Arthritis
Patients. Rheumatol Ther.

[B13] Keystone EC, Breedveld FC, Kupper H, Li Y, Florentinus S, Sainsbury I (2018). Long-term use of adalimumab as monotherapy after attainment of
low disease activity with adalimumab plus methotrexate in patients with
rheumatoid arthritis. RMD Open.

[B14] Schaible TF (2000). Long term safety of infliximab. Can J Gastroenterol.

[B15] Fasth AER, Cao D, Van Vollenhoven R, Trollmo C, Malmström V (2004). CD28nullCD4+ T cells--characterization of an effector memory
T-cell population in patients with rheumatoid arthritis. Scand J Immunol.

[B16] Chandy KG, Cahalan M, Pennington M, Norton RS, Wulff H, Gutman GA (2001). Potassium channels in T lymphocytes: toxins to therapeutic
immunosuppressants. Toxicon.

[B17] Panyi G, Varga Z, Gáspár R (2004). Ion channels and lymphocyte activation. Immunol Lett.

[B18] Ghanshani S, Wulff H, Miller MJ, Rohm H, Neben A, Gutman GA, Cahalan MD, Chandy KG (2000). Up-regulation of the IKCa1 Potassium Channel during T-cell
Activation. J Biol Chem.

[B19] Oliveira IS, Ferreira IG, Alexandre-Silva GM, Cerni FA, Cremonez CM, Arantes EC, Zottich U, Pucca MB (2019). Scorpion toxins targeting Kv1.3 channels: Insights into
immunosuppression. J Venom Anim Toxins incl Trop Dis.

[B20] Feske S, Skolnik EY, Prakriya M (2012). Ion channels and transporters in lymphocyte function and
immunity. Nat Rev Immunol.

[B21] Casella-Martins A, Ayres LR, Burin SM, Morais FR, Pereira JC, Faccioli LH, Sampaio SV, Arantes EC, Castro FA, Pereira-Crott LS (2015). Immunomodulatory activity of Tityus serrulatus scorpion venom on
human T lymphocytes. J Venom Anim Toxins incl Trop Dis.

[B22] Sands SB, Lewis RS, Cahalan MD (1989). Charybdotoxin blocks voltage-gated K+ channels in human and
murine T lymphocytes. J Gen Physio.

[B23] Price M, Lee SC, Deutsch C (1989). Charybdotoxin inhibits proliferation and interleukin 2 production
in human peripheral blood lymphocytes. Proc Natl Acad Sci U S A.

[B24] Giangiacomo KM, Garcia ML, McManus OB (1992). Mechanism of iberiotoxin block of the large-conductance
calcium-activated potassium channel from bovine aortic smooth
muscle. Biochemistry.

[B25] Valverde P, Kawai T, Taubman MA (2004). Selective blockade of voltage-gated potassium channels reduces
inflammatory bone resorption in experimental periodontal
disease. J Bone Miner Res.

[B26] Garcia-Calvo M, Leonard RJ, Novick J, Stevens SP, Schmalhofer W, Kaczorowski GJ, Garcia ML (1993). Purification, characterization, and biosynthesis of margatoxin, a
component of Centruroides margaritatus venom that selectively inhibits
voltage-dependent potassium channels. J Biol Chem.

[B27] Mouhat S, Visan V, Ananthakrishnan S, Wulff H, Andreotti N, Grissmer S, Darbon H, De Waard M, Sabatier JM (2005). K+ channel types targeted by synthetic OSK1, a toxin from
Orthochirus scrobiculosus scorpion venom. Biochem J.

[B28] Gurrola GB, Hernández-López RA, Rodríguez de la Vega RC, Varga Z, Batista CVF, Salas-Castillo SP, Panyi G, del Río-Portilla F, Possani LD (2012). Structure, Function, and Chemical Synthesis of Vaejovis mexicanus
Peptide 24: A Novel Potent Blocker of Kv1.3 Potassium Channels of Human T
Lymphocytes. Biochemistry.

[B29] Hashemlou M, Zare Mirakabadi A, Ahmadi M, Hejazi M (2009). Study on anti inflammatory effect of scorpion (Mesobuthus eupeus)
venom in adjuvant-induced arthritis in rats. Arch Razi Inst.

[B30] Tanner MR, Pennington MW, Chamberlain BH, Huq R, Gehrmann EJ, Laragione T, Gulko PS, Beeton C (2018). Targeting KCa1.1 channels with a scorpion venom peptide for the
therapy of rat models of rheumatoid arthritis. J Pharmacol Exp Ther.

[B31] Zoccal KF, Bitencourt C da S, Secatto A, Sorgi CA, Bordon K de CF, Sampaio SV, Candiani E, Faccioli LH (2011). Tityus serrulatus venom and toxins Ts1, Ts2, and Ts6 induce
macrophage activation and production of immune mediators. Toxicon.

[B32] Petricevich VL, Lebrun I (2005). Immunomodulatory effects of the Tityus serrulatus venom on murine
macrophage functions in vitro. Mediators Inflamm.

[B33] Adi-Bessalem S, Hammoudi-Triki D, Laraba-Djebari F (2008). Pathophysiological effects of Androctonus australis hector
scorpion venom: Tissue damages and inflammatory response. Exp Toxicol Pathol.

[B34] Morales Duque H (2013). Caracterização de novos peptídeos bloqueadores de canais para K+
isolados da peçonha do escorpião Tityus sp. Repos Inst da UNB.

[B35] Lowe RM, Farrell PM (2011). A portable device for the electrical extraction of scorpion
venom. Toxicon.

[B36] Page B, Page M, Noel C (1993). A new fluorometric assay for cytotoxicity measurements
in-vitro. Int J Oncol.

[B37] O’Brien J, Wilson I, Orton T, Pognan F (2000). Investigation of the Alamar Blue (resazurin) fluorescent dye for
the assessment of mammalian cell cytotoxicity. Eur J Biochem.

[B38] Solomon DH, Bitton A, Katz JN, Radner H, Brown EM, Fraenkel L (2014). Review: treat to target in rheumatoid arthritis: fact, fiction,
or hypothesis?. Arthritis Rheumatol (Hoboken, NJ).

[B39] Aletaha D, Neogi T, Silman AJ, Funovits J, Felson DT, Bingham CO, Birnbaum NS, Burmester GR, Bykerk VP, Cohen MD, Combe B, Costenbader KH, Dougados M, Emery P, Ferraccioli G, Hazes JM, Hobbs K, Huizinga TW, Kavanaugh A, Kay J, Kvien TK, Laing T, Mease P, Ménard HA, Moreland LW, Naden RL, Pincus T, Smolen JS, Stanislawska-Biernat E, Symmons D, Tak PP, Upchurch KS, Vencovský J, Wolfe F, Hawker G (2010). 2010 Rheumatoid arthritis classification criteria: An American
College of Rheumatology/European League Against Rheumatism collaborative
initiative. Arthritis Rheum.

[B40] Lyons AB (2000). Analysing cell division in vivo and in vitro using flow
cytometric measurement of CFSE dye dilution. J Immunol Methods.

[B41] Petrelli A, Van Wijk F (2016). CD8+ T cells in human autoimmune arthritis: The unusual
suspects. Nat Rev Rheumatol.

[B42] Carvalheiro H, Duarte C, Silva-Cardoso S, da Silva JAP, Souto-Carneiro MM (2015). CD8+ T cell profiles in patients with rheumatoid arthritis and
their relationship to disease activity. Arthritis Rheumatol.

[B43] Kindt T, Goldsby R, Osborne B, McGraw-Hill Interamericana (2007). Inmunologia de Kuby.

[B44] Lam J, Wulff H (2011). The lymphocyte potassium channels Kv1.3 and KCa3.1 as targets for
immunosuppression. Drug Dev Res.

[B45] Varga Z, Gurrola-Briones G, Papp F, Rodriguez De La Vega RC, Pedraza-Alva G, Tajhya RB, Gaspar R, Cardenas L, Rosenstein Y, Beeton C, Possani LD, Panyi G (2012). Vm24, a natural immunosuppressive peptide, potently and
selectively blocks Kv1.3 potassium channels of human T cells. Mol Pharmacol.

[B46] Wulff H, Calabresi PA, Allie R, Yun S, Pennington M, Beeton C, Chandy KG (2003). The voltage-gated Kv1.3 K+ channel in effector memory T cells as
new target for MS. J Clin Invest.

[B47] Hu L, Pennington M, Jiang Q, Whartenby KA, Calabresi PA (2007). Characterization of the functional properties of the
voltage-gated potassium channel Kv1.3 in human CD4+ T
lymphocytes. J Immunol.

[B48] Arenas CJ (2013). Identificação e caracterização de peptídeos moduladores de canais para
sódio presentes na peçonha do escorpião tityus sp. pertencente ao grupo
forcipula.

[B49] Barona J, Batista CVF, Zamudio FZ, Gomez-Lagunas F, Wanke E, Otero R, Possani LD (2006). Proteomic analysis of the venom and characterization of toxins
specific for Na+- and K+-channels from the Colombian scorpion Tityus
pachyurus. Biochim Biophys Acta.

[B50] Guerrero-Vargas JA, Mourão CBF, Quintero-Hernández V, Possani LD, Schwartz EF (2012). Identification and phylogenetic analysis of Tityus pachyurus and
Tityus obscurus novel putative Na +-channel scorpion toxins. PLoS One.

[B51] Guerrero-Vargas JA, da Silva Libério M, Souza Castro M, Asociación Colombiana para el avance de la ciencia (2008). Colombia, Innovación Y Ciencia.

[B52] Wang X, Li G, Guo J, Zhang Z, Zhang S, Zhu Y, Cheng J, Yu L, Ji Y, Tao J (2020). Kv1.3 Channel as a Key Therapeutic Target for Neuroinflammatory
Diseases: State of the Art and Beyond. Front Neurosci.

[B53] Zhao Y, Huang J, Yuan X, Peng B, Liu W, Han S, He X (2015). Toxins targeting the Kv1.3 Channel: Potential immunomodulators
for autoimmune diseases. Toxins (Basel).

[B54] Rashid MH, Huq R, Tanner MR, Chhabra S, Khoo KK, Estrada R, Dhawan V, Chauhan S, Pennington MW, Beeton C, Kuyucak S, Norton RS (2014). A potent and Kv1.3-selective analogue of the scorpion toxin HsTX1
as a potential therapeutic for autoimmune diseases. Sci Rep.

[B55] Corzo G, Espino-Solis GP (2017). Selected scorpion toxin exposures induce cytokine release in
human peripheral blood mononuclear cells. Toxicon.

[B56] Lebrun B, Romi-Lebrun R, Martin-Eauclaire MF, Yasuda A, Ishiguro M, Oyama Y, Pongs O, Nakajima T (1997). A four-disulphide-bridged toxin, with high affinity towards
voltage-gated K+ channels, isolated from Heterometrus spinnifer
(Scorpionidae) venom. Biochem J.

[B57] Veytia-Bucheli JI, Jiménez-Vargas JM, Melchy-Pérez EI, Sandoval-Hernández MA, Possani LD, Rosenstein Y (2018). K v 1.3 channel blockade with the Vm24 scorpion toxin attenuates
the CD4 + effector memory T cell response to TCR stimulation. Cell Commun Signal.

[B58] Wang X, Dong L, Liang Y, Ni H, Tang J, Xu C, Zhou Y, Su Y, Wang J, Chen D, Mao C (2015). Performance evaluation of FlowCytomix assays to quantify
cytokines in patients with rheumatoid arthritis. Int J Clin Exp Med.

[B59] McInnes IB, Schett G (2007). Cytokines in the pathogenesis of rheumatoid
arthritis. Nat Rev Immunol.

